# Acetylcholine acts through M3 muscarinic receptor to activate the EGFR signaling and promotes gastric cancer cell proliferation

**DOI:** 10.1038/srep40802

**Published:** 2017-01-19

**Authors:** Huangfei Yu, Hongwei Xia, Qiulin Tang, Huanji Xu, Guoqing Wei, Ying Chen, Xinyu Dai, Qiyong Gong, Feng Bi

**Affiliations:** 1 Department of Medical Oncology and Laboratory of Molecular Targeted Therapy in Oncology, State Key Laboratory of Biotherapy, West China Hospital, Sichuan University, Chengdu 610041, Sichuan, China; 2 Department of Radiology, West China Hospital, Sichuan University, Chengdu 610041, Sichuan, China

## Abstract

Acetylcholine (ACh), known as a neurotransmitter, regulates the functions of numerous fundamental central and peripheral nervous system. Recently, emerging evidences indicate that ACh also plays an important role in tumorigenesis. However, little is known about the role of ACh in gastric cancer. Here, we reported that ACh could be auto-synthesized and released from MKN45 and BGC823 gastric cancer cells. Exogenous ACh promoted cell proliferation in a does-dependent manner. The M3R antagonist 4-DAMP, but not M1R antagonist trihexyphenidyl and M2/4 R antagonist AFDX-116, could reverse the ACh-induced cell proliferation. Moreover, ACh, via M3R, activated the EGFR signaling to induce the phosphorylation of ERK1/2 and AKT, and blocking EGFR pathway by specific inhibitor AG1478 suppressed the ACh induced cell proliferation. Furthermore, the M3R antagonist 4-DAMP and darifenacin could markedly inhibit gastric tumor formation *in vivo*. 4-DAMP could also significantly enhance the cytotoxic activity of 5-Fu against the MKN45 and BGC823 cells, and induce the expression of apoptosis-related proteins such as Bax and Caspase-3. Together, these findings indicated that the autocrine ACh could act through M3R and the EGFR signaling to promote gastric cancer cells proliferation, targeting M3R or EGFR may provide us a potential therapeutic strategy for gastric cancer treatment.

Gastric cancer is the second common cause of cancer-related death worldwide^1^,^2^. Although the incidence and mortality rate of gastric cancer have declined in recent decades, there are still many gastric cancer patients diagnosed at an advanced stage and died within 2 years despite surgery or other treatments^3^. Thus, it’s very urgent to find novel mechanism that contribute to gastric cancer progression. The mechanism would not only further our insight in gastric carcinogenesis, but also provide us new effective strategies for gastric cancer treatment.

Acetylcholine, an important neurotransmitter, is a key mediator in the central and peripheral nervous systems, and plays critical roles in learning, memory, autonomic control and muscular contraction^4^,^5^,^6^,^7^. Many studies recently have revealed that ACh also plays a non-neuronal role in some physiological and pathological process including inflammatory diseases^8^,^9^, functional bowel disorders^10^,^11^, as well as several kinds of cancer^12^,^13^. Several reports have indicated that ACh could act as a potential growth factor to stimulate cancer cell proliferation in lung cancer^14^, breast cancer^15^, colon cancer^16^ *et al*. Furthermore, ACh could also be synthesized and considered as auto-stimulating growth factor in some types of cancer^17^,^18^. Very recently, Wang *et al*. reported that the M3 muscarinic receptor mediated ACh-induced proliferation in gastric cancer cells^19^. However, it’s still unclear about the concrete non-neuronal role of ACh in gastric carcinogenesis.

Here, we showed that there existed an autocrine-loop for ACh in gastric cancer cells, and ACh could act as a growth factor to promote cell proliferation. Activation of M3R and EGFR pathway might be a potential mechanism for ACh induced cell growth. And M3R antagonism 4-DAMP combined with 5-Fluorouracil (5-Fu) could significantly reduce the cell viability and enhance apoptosis in MKN45 and BGC823 gastric cancer cells.

## Results

### ChAT protein expression in gastric cancer cells

**Figure 1:**
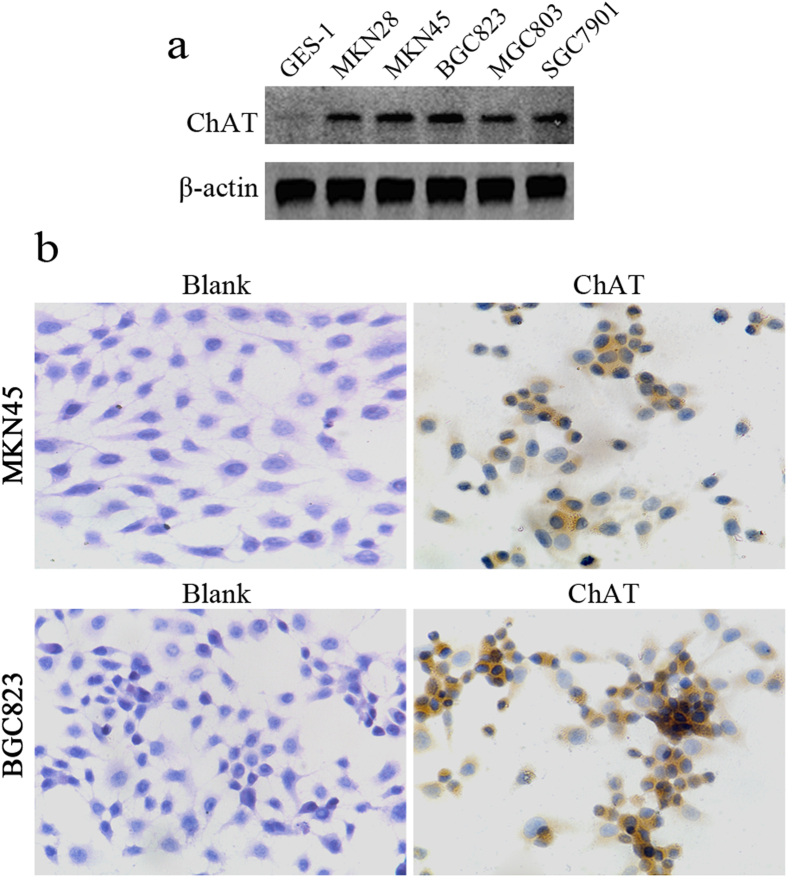
ChAT expression in gastric cancer cell lines and normal gastric epithelial cells. (**a**) The expression of ChAT was analyzed by western blot in gastric cancer cell lines MKN28, MKN45, BGC823, MGC803, SGC7901 and normal gastric epithelial cells GES-1. (**b**) Immunohistochemical analysis showed ChAT expression in MKN45 and BGC823 cells. Original magnification ×200.

Firstly, we determined whether ChAT, the rate-limiting enzyme that synthesize ACh, were expressed in different human gastric cancer cell lines and normal gastric epithelial cells. As shown in Fig. 1a, ChAT protein was differentially expressed in human gastric cell lines, it was strongly expressed in MKN28, MKN45, BGC823, MGC803, and SGC7901 gastric cancer cells, while weak positive in normal gastric epithelial cells (GES-1). Immunohistochemistry staining was further to verify the ChAT expression in MKN45 and BGC823 cells, the results showed that ChAT was over-expressed and mainly located in the cytoplasm of the two cell lines (Fig. 1b).

### ACh secretion in human gastric cancer cells

**Figure 2:**
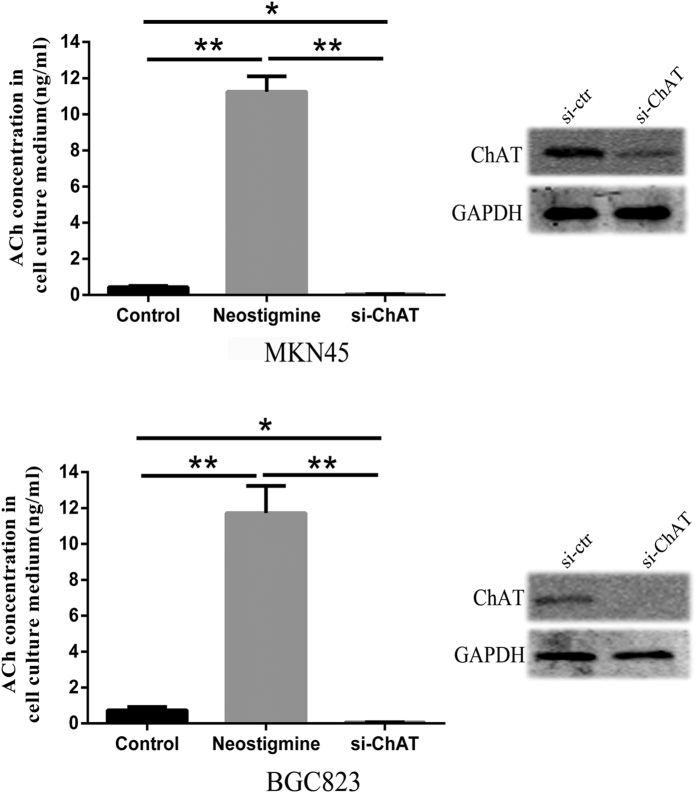
ACh released from MKN45 and BGC823 cells. (**a**) ACh concentration in the MKN45 and BGC823 (**b**) cell culture media. Neostigmine was added at a concentration of 50 μM after plating and cells were incubated for 24 h, while ChAT protein in cells were knockdown by transfected with siRNA for 48 h, then the serum-free media were removed for LC-MS/MS assay of ACh, and the knockdown efficiency was verified by western blot. (*P < 0.05; **P < 0.01).

As ChAT is expressed in different human GC cell lines, the synthesized ACh might be released into the culture medium. Further LC-MSMS analysis showed that the concentration of ACh were about 0.44 ± 0.06 ng/ml and 0.72 ± 0.19 ng/ml in serum-free cell culture medium of MKN45 and BGC823 respectively. And the concentration of ACh were dramatically increased to 10.91 ± 1.33 ng/ml and 11.72 ± 1.52 ng/ml respectively (*p* < 0.01) with addition of the acetylcholinesterase inhibitor neostigmine in the two cell lines. While the ACh concentration could hardly be measured in the culture medium of MKN45 and BGC823 cell with ChAT knockdown (Fig. 2a,b). Together, these data indicated that the gastric cancer cells could synthesize and secret ACh through ChAT.

### ACh promotes cell proliferation and stimulates phosphorylation of ERK and AKT in gastric cancer cells

**Figure 3:**
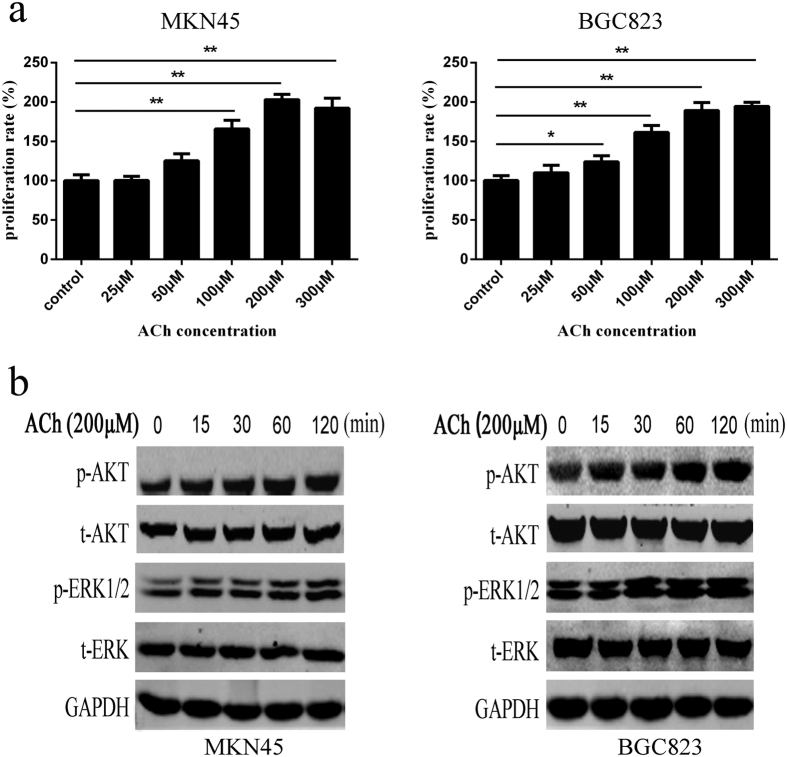
ACh promotes cell proliferation and stimulates phosphorylation of ERK1/2 and AKT in MKN45 and BGC823 cells. (**a**) MKN45 and BGC823 cells were incubated with ACh for 72 h at indicated concentrations. CCK-8 assay was used to determine the proliferation of cancer cells. (**b**) Western blot was performed to show expression changes of the indicated proteins after treating cells with 200 μM ACh for 15 min to 120 min.(*P < 0.05; **P < 0.01).

To explore the biological function of ACh in human gastric cancer cells, firstly, we verified that all of five muscarinic ACh receptor subtypes were expressed in different gastric cancer cell lines (Supplementary Fig. 1a), and the basic concentration of endogenous ACh was also explored in different cell culture medium. MKN45 and BGC823 cells was selected to study the effect of exogenous ACh on the cell proliferation because of their relatively low levels of endogenous ACh (Supplementary Fig. 1b). As shown in Fig. 3a, ACh could promote cell proliferation in a does-dependent manner varying from the concentration of 50 μM to 300 μM, and it reached peak at concentration of 200 μM. We then investigated the expression of p-ERK1/2 and p-AKT induced by ACh, and found that 200 μM ACh stimulation enhanced the phosphorylation level of p-ERK1/2 and p-AKT from 15 min to 2 h (Fig. 3b). We also found that exogenous ACh have similar effects in promoting cell proliferation and stimulating the phosphorylation level of p-ERK1/2 and p-AKT in SGC7901 cells which have the lowest endogenous ACh (Supplementary Fig. 2a,b). These results suggested that exogenous ACh stimulation could promote cell proliferation and increase phosphorylation of ERK and AKT in gastric cancer cells.

### M3 AChR mediates ACh-induced cell proliferation and phosphorylation of ERK1/2 and AKT

**Figure 4:**
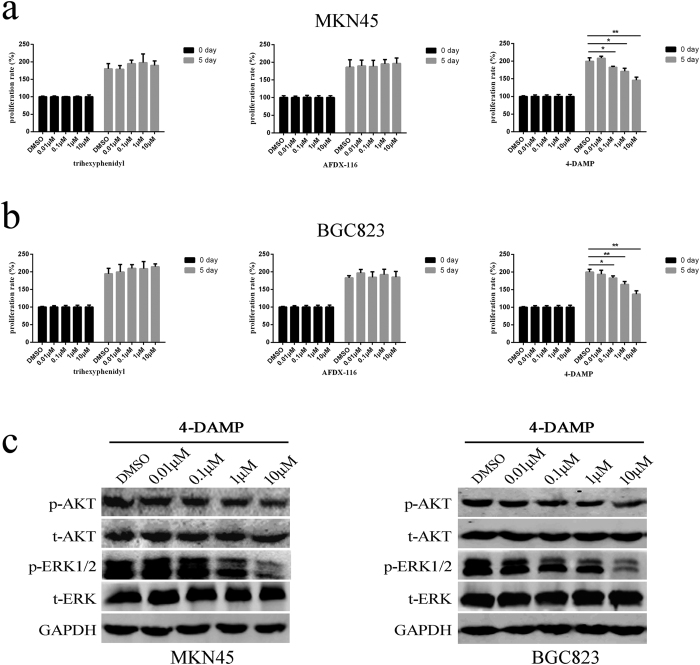
M3 AChR mediates endogenous ACh-induced cell proliferation and phosphorylation of ERK1/2 and AKT. After cells were treated with trihexyphenidyl (M1R antagonist), AFDX-116 (M2R/M4R antagonist), and 4-DAMP (M3R antagonists) for 5 days at indicated concentrations, CCK-8 assay was used to detect cell growth in MKN45 (**a**) and BGC823 cells (**b**). (**c**) Western blot showed the expression changes of p-ERK1/2 and p-AKT in MKN45 and BGC823 cells under 0.01 μM to 10 μM of 4-DAMP treatment for 4 h. (*P < 0.05; **P < 0.01).

**Figure 5:**
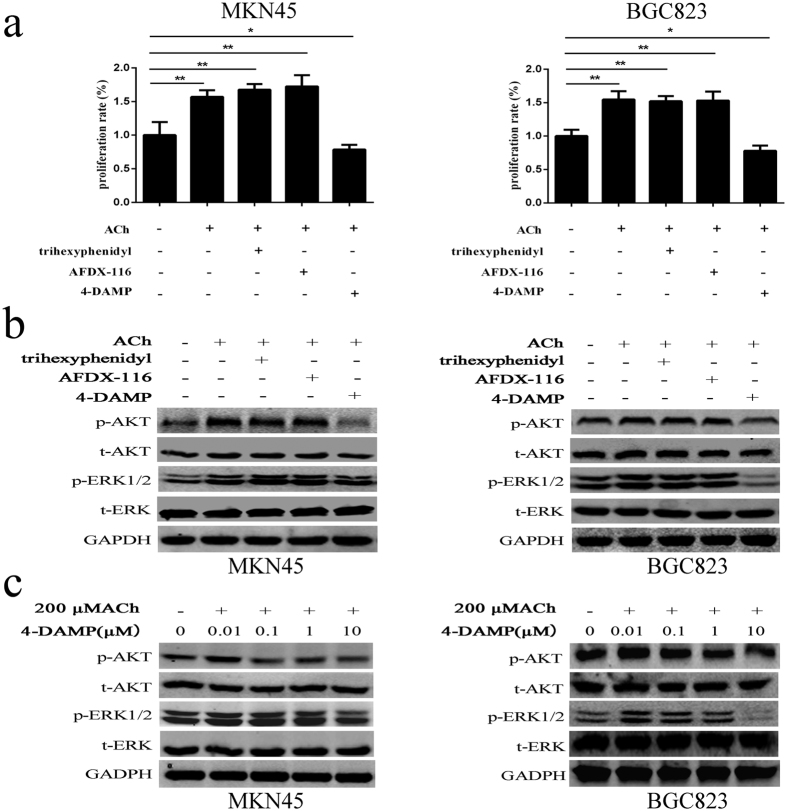
M3 AChR mediates the effects of exogenous ACh on promoting cell growth and activating p-ERK1/2 and p-AKT. (**a**) Inhibitors for M1R (trihexyphenidyl), M2R/M4R (AFDX-116) and M3R (4-DAMP) were added in culture medium 4 h before 200 μM ACh addition, CCK-8 assay was used to determine which muscarinic receptor mainly mediates the effect of exogenous ACh on cell growth. (**b)** Western blot was performed to analyze which muscarinic receptor mediates the ACh-induced phosphorylation of ERK1/2 and AKT. (**c**) Western blot showed the expression changes of phosphorylated ERK1/2 and AKT in cells under 0.01 μM~10 μM concentrations of 4-DAMP treatment for 4 h prior to 200 μM ACh addition. (*P < 0.05; **P < 0.01).

ACh mainly acts through muscarinic receptors to transduct extracellular signals in tumorigenesis^20^,^21^. As shown in the above data, both M1~M5 muscarinic receptor were expressed in MKN45 and BGC823 cells. To identify which subtype mainly mediate ACh induced gastric cancer cells proliferation, we firstly used the antagonists of M1~4 receptor separately to inhibit the effect of endogenous ACh in MKN45 and BGC823. As shown in Fig. 4a,b, M3 selective antagonist 4-DAMP markedly inhibited cell proliferation at 5 days, whereas the selective M1 antagonist trihexyphenidyl and the M2/M4 selective antagonist AFDX-116 have nearly no effect on the gastric cancer cell proliferation. The inhibition effect was also observed when cells were transfected with M3R siRNA (Supplementary Fig. 3a,b). 4-DAMP alone could dramatically inhibited the expression of p-ERK1/2 and p-AKT in MKN45 and BGC823 cells (Fig. 4c). In addition, exogenous ACh induced cell proliferation can be reversed by 4-DAMP, but not the selective M1 antagonist trihexyphenidyl and the M2/M4 selective antagonist AFDX-116 (Fig. 5a). At protein levels, only the 4-DAMP, but not trihexyphenidyl and AFDX-116, could reverse the ACh induced phosphorylation of ERK1/2 and AKT (Fig. 5b,c). These results suggest that M3R plays an crucial role in the endogenous and exogenous ACh-induced cell proliferation and phosphorylation of ERK and AKT in gastric cancer cells.

### ACh acts through M3R to activate EGFR signaling and promotes cell proliferation in gastric cancer cells

**Figure 6:**
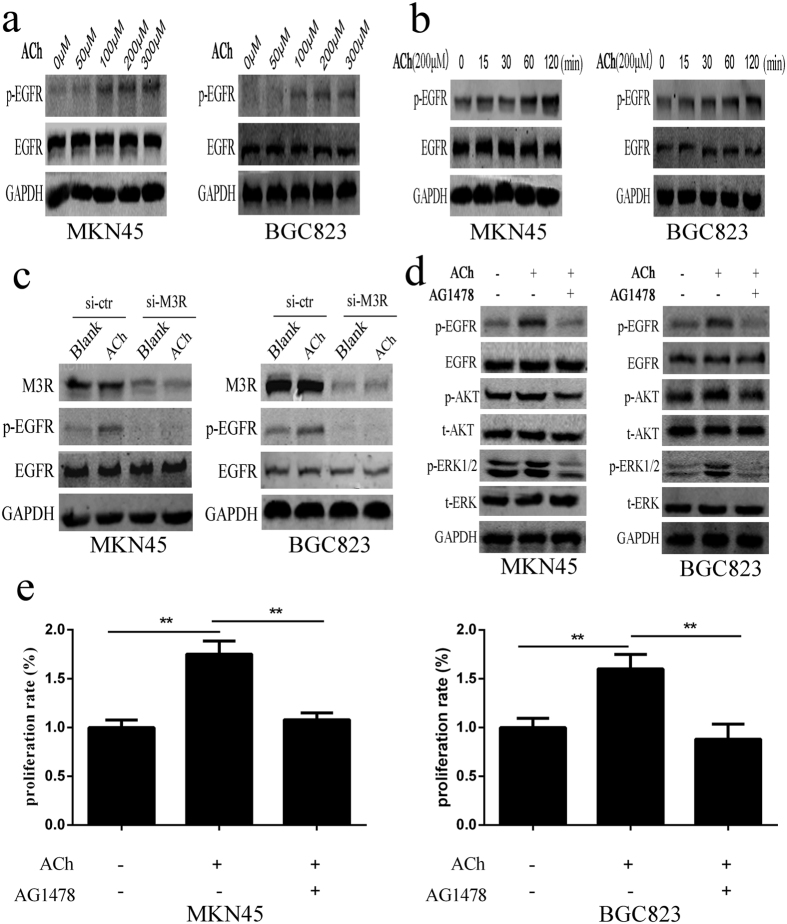
EGFR participates the process of ACh induced cell proliferation and signaling transduction. (**a**) Cells were treated with indicated concentrations of ACh for 2 h, EGFR and phosphorylated EGFR were analyzed by western blot. (**b**) Cells were treated with 200 μM ACh for 15 min to 120 min, EGFR and phosphorylated EGFR were analyzed by western blot. (**c**) After knockdown of M3 AChR expression by transfecting small interfering RNA, p-EGFR expression stimulated by ACh were analyzed by western blot in MKN45 and BGC823 cells. (**d**) After adding 10 μM EGFR specific inhibitor AG1478 2 h prior to ACh addition, protein changes were analyzed by western blot. (**e**) AG1478 was added 2 h before ACh addition, CCK-8 assay was used to study the role of EGFR in ACh induced cell proliferation. (**P < 0.01).

Previous studies have indicated that the G protein coupled receptor (GPCR) family members could trans-activate EGFR signaling, thus induced the phosphorylation of ERK1/2 and AKT^22^. Our above-mentioned data showed that ACh could act through M3R to induce the phosphorylation of ERK1/2 and AKT, while the M3R belongs to the GPCR members. So we then investigated whether ACh could act through M3R to activate EGFR signaling in gastric cancer cells. Indeed, the p-EGFR was significantly increased with ACh stimulation (Fig. 6a). Simultaneously, p-EGFR was also enhanced by ACh in a time-dependent manner from 15 min to 2 h (Fig. 6b). To determine whether M3R participated in the transactivation of EGFR, cells were transfected with M3R siRNA (siM3R) or Control siRNA (siCtrl) for 72 h and then stimulated with ACh for 2 h. As shown in Fig. 6c, the ACh induced activation of EGFR was significantly reversed with M3R knockdown, and these data indicated that ACh could act through M3R to activate EGFR. We further used AG1478, a specific EGFR inhibitor to study its functional role in ACh induced cell proliferation, and the results showed that ACh induced phosphorylation of EGFR, ERK1/2 and AKT could be reversed by 10 μM AG1478 (Fig. 6d). AG1478 can also counteract ACh induced cell proliferation in MKN45 and BGC823 cells (Fig. 6e). Moreover, we also used the ERK specific inhibitor U0126 and the AKT specific inhibitor MK2206 to further investigate their roles in ACh-induced proliferation, and found that the enhancement of p-ERK and p-AKT stimulated by ACh could be reversed by U0126 and MK2206 respectively (Supplementary Fig. 4a,b). U0126 and MK2206 could also block the proliferation induced by ACh stimulation in MKN45 and BGC823 cells (Supplementary Fig. 4c). The above data indicated that ACh could act through M3R to activate EGFR signaling and promote cell proliferation in gastric cancer cells.

### The M3R inhibitors inhibit gastric tumor formation

**Figure 7:**
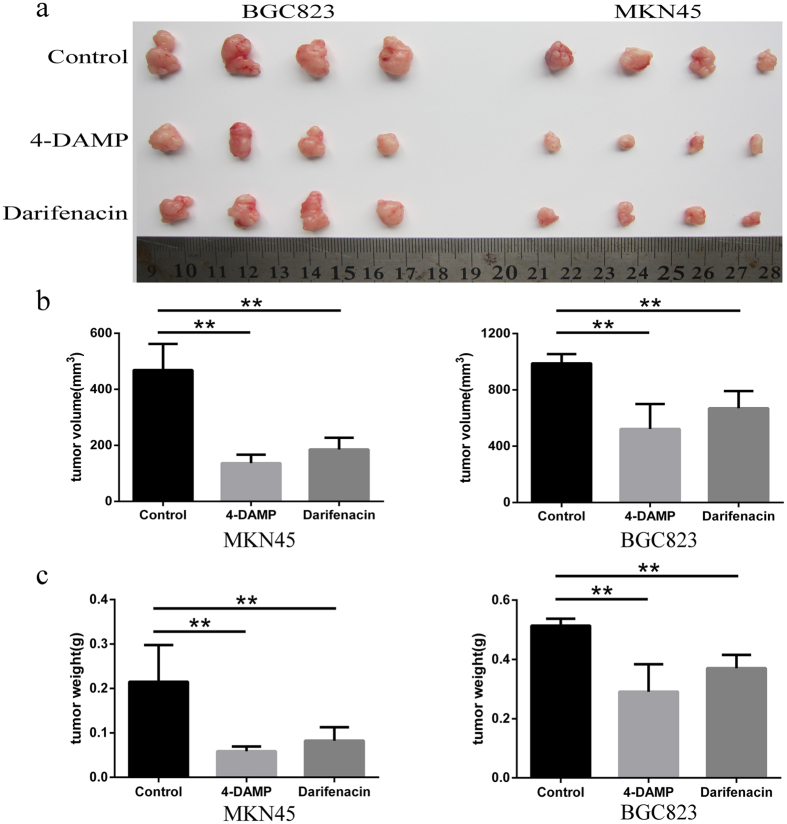
M3R antagonist 4-DAMP and darifenacin inhibit gastric xenograft tumor growth. (**a**) After 12 days treated by 4-DAMP and darifenacin, the general morphology and tumor size of xenografts in MKN45 and BGC823 group were measured. (**b**) Tumor volume of each group in MKN45 and BGC823 xenografts were quantitated. (**c**) Tumor weight of each group in MKN45 and BGC823 xenografts were measured. (**P < 0.01).

To further explore the function of M3R in gastric cancer growth *in vivo*, two M3R antagonist, 4-DAMP and darifenacin, were used to investigated the potential role of M3R in tumor xenografts growth in nude mice. As shown in Fig. 7a, tumor sizes were dramatically reduced in both MKN45 and BGC823 xenografts with 12 days treatment of 4-DAMP or darifenacin,. The tumor volumes were 468.76 ± 93.44 mm^3^ and 988.58 ± 65.69 mm^3^ in MKN45 and BGC823 control groups, which had reduced to 136.31 ± 30.51 mm^3^ and 522.13 ± 177.17 mm^3^ respectively by treating with 4-DAMP, and these data in darifenacin groups were 185.45 ± 41.67 mm^3^ and 669.38 ± 122.90 mm^3^ respectively (Fig. 7b) (both *p* < 0.01). The tumor weights of gastric cancer xenografts in 4-DAMP and darifenacin group have the same change tendency as tumor volume. The weights were 0.22 ± 0.08 g and 0.51 ± 0.02 g in MKN45 and BGC823 control group, and they had significantly decreased to 0.06 ± 0.01 g and 0.29 ± 0.09 g in 4-DAMP group and 0.08 ± 0.03 g and 0.37 ± 0.05 g in darifenacin group respectively (Fig. 7c) (both *p* < 0.01). Those results indicated that M3R played a crucial role in gastric tumorigenesis and blocking M3R function could impede gastric cancer growth *in vivo*.

### The M3R inhibitor 4-DAMP could increase the sensitivity of gastric cancer cells to 5-Fu

**Figure 8:**
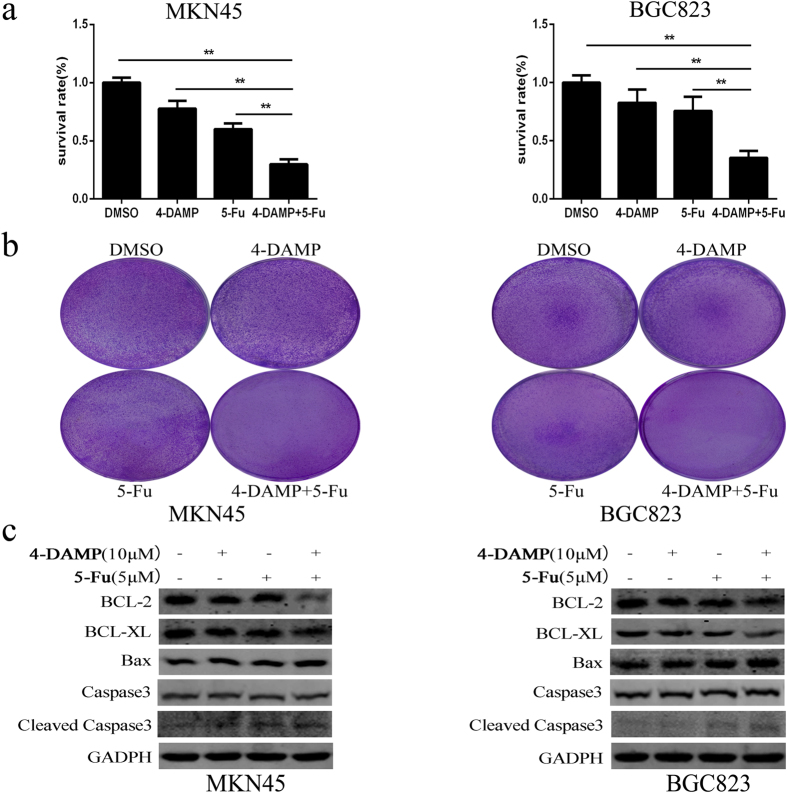
Combined use of 4-DAMP with 5-Fu can inhibit cell growth and promote apoptosis in MKN45 and BGC823 cells. (**a**) CCK-8 assay was performed to analyze cell viability in 4-DAMP alone, 5-Fu alone, and 4-DAMP plus 5-Fu combined group. (**b**) The clone formations of indicated group were determined by plate clone assay. (**c**) Apoptosis-related proteins were analyzed by western blot. (**P < 0.01).

The above data indicated that M3 muscarinic receptor was essential for ACh induced cell proliferation *in vitro* and *in vivo*, so we next studied whether the M3R inhibitor 4-DAMP could improve the cytotoxic effect of chemotherapeutic drugs. Firstly, we examined the IC50 value of a common chemotherapy drug 5-Fu in the gastric cancer cell lines, the data showed that MKN45 and BGC823 cells had the highest IC50 among all the cell lines, which the IC50 value were 17.0 μg/ml and 6.95 μg/ml respectively (Supplementary Fig. 5). Combination of 10 μM 4-DAMP and 5 μM 5-Fu reduced the cell viability to (30 ± 4.0)% and (35 ± 6.0)% in MKN45 and BGC823 cells respectively, which had significant difference when compared with the 4-DAMP and 5-FU alone group (*p* < 0.01) (Fig. 8a). The results of colony formation were consistent with the data of CCK-8 assay (Fig. 8b). We further analyzed the expression of apoptosis-related proteins, and found that Bcl-xl and Bcl-2 were significantly downregulated, while Bax and cleaved caspase-3 were notablely up-regulated in combined group when compared with control group and 4-DAMP or 5-FU alone group (Fig. 8c). Overall, these data demonstrated that M3R inhibitor 4-DAMP could enhance the chemo-sensitivity of 5-Fu and induce apoptosis-related proteins expression in gastric cancer cells.

## Discussion

In some cases, cancer cells can grow around the nerves and eventually invade them, and this process was defined as perineural invasion which plays an important role in tumor progression in many cancer types^23^,^24^. During perineural invasion, nerves are passive and provide a route for cancer cell dissemination. However, little is known about the role of nerve and neurotransmitter on tumor development and progression^25^.

**Figure 9:**
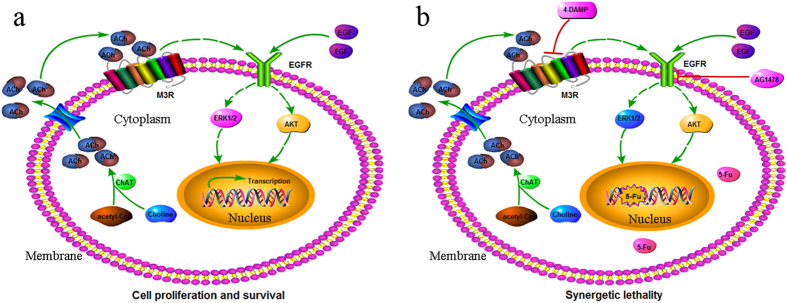
Schematic representation of the proposed mechanism of ACh-modulated M3/EGFR pathway in gastric cancer. (**a**) In gastric cancer cells, cytoplasm ChAT protein catalyzes ACh synthesis, then ACh is secreted and released into the extracellular space, the extracellular ACh could combine and activate M3 receptor located in cytomembrane of cells, following activating EGFR signaling, and then leading to the increasement of p-ERK1/2 and p-AKT, thus, promote gastric cancer cell proliferation and survival. (**b**) When the function of M3R was blocked by specific antagonists (such as 4-DAMP), or its downstream effector EGFR, inhibited by specific inhibitor AG1478, the auto-stimulating role of ACh in cell proliferation will be weaken in some degree. The M3R/EGFR signaling inhibitors can enhance the chemosensitivity of 5-Fu and reduce cell viability in gastric cancer. The combined strategy may provide a feasible strategy for gastric cancer therapy.

Recently, Magnon and colleagues reported that autonomic sympathetic and parasympathetic nerve sprouting is essential for cancer progression in mice prostate tumors, norepinephrine and acetylcholine, the neurotransmitter of sympathetic and parasympathetic nerve could stimulate prostate cancer growth and metastasis^26^. Subsequently, Zhao *et al*.^27^ showed that surgical or pharmacological denervation of the stomach strongly reduced tumor incidence and progression of gastric cancer, and was able to enhance the therapeutic effect of systemic chemotherapy. Those researches strongly imply that the neurotransmitters ACh might play an important role in tumor progression. In the current study, we showed that ACh could also promote the proliferation of gastric cancer cells. Indeed, ACh can be auto-synthesized and released as a cell growth promoter in gastric cancer cells, and the autocrine loop of ACh is indeed existed in these cells (Fig. 9a). Our data was also consistent with Wang *et al*.’s study which indicated the proliferation-promoting role of ACh in gastric cancer cells^19^.

Many studies have demonstrated that ACh mainly acts through muscarinic receptors to transduce the exo-cellular signaling and plays an critical role in tumorigeneis^28^,^29^. The muscarinic receptors contained five subtypes, including M1~5 receptors. Our results have shown that M3 subtype, but not other muscarinic receptors, mediated the cell proliferation stimulated by exogenous or endogenous ACh. The M3 receptor, which selectively conjugate to the G proteins of the Gq family, could transactivate several second messengers and modulate numerous fundamental functions in central and peripheral nervous system^30^,^31^. Recent years, increasing evidences demonstrated that M3R may play a vital role in the carcinogenesis of many types of cancer, including colon cancer, lung cancer, and cholangiocarcinoma^32^,^33^,^34^. Notably, in human gastric cancer tissue, M3R was over-expressed and correlated with the cancer stage and lymph node metastasis^19^. Those studies, combined with our preliminary data suggested that M3R plays a major role in ACh stimulated gastric cell proliferation.

We have demonstrated that ACh could act though M3R to activate EGFR pathway, while knockdown M3R or inhibiting EGFR could reverse the cell proliferation and phosphorylation of ERK and AKT induced by ACh stimulation in gastric cancer. The data suggested that EGFR was an indispensable molecular in the process of ACh stimulated gastric cancer cell proliferation. The exact mechanism between M3R and EGFR is still unclear in gastric cancer. Studies in colon cancer revealed that M3R was able to promote MMP7 to catalyze the release of HB-EGF and thus facilitate ACh-induced EGFR signaling activation^35^. However, whether the same mechanism still works in gastric cancer need further investigation.

The present data demonstrated that blocking M3R function could not only inhibit gastric cancer growth *in vitro* and *in vivo*, but also enhance cytotoxicity of 5-Fu against gastric cancer cells, implying that M3R may be as a potential target for gastric cancer. Additionally, M3R functional inhibition by small interference RNA also contributed to apoptosis and restrained cell invasion/migration in gastric cancer^36^. Song *et al*. reported that inhibiting the function of M3R could suppress tumor growth in nude mice suffering small cell lung carcinoma xenografts^37^. Those results together with our’s suggested that targeting M3R was probably a new therapeutic strategy in gastric cancer treatment.

In all, the current study indicated that acetylcholine could act through M3 muscarinic receptor to activate the EGFR signaling and promote gastric cancer cell proliferation. Blocking the function of M3R could not only switch off the intracellular signaling transduction of ACh for stimulating cell growth, but also potentiate cytotoxic activity of 5-Fu against gastric cancer (Fig. 9b). These findings give us further insight in the gastric carcinogenesis and provide new strategies for gastric cancer therapy.

## Materials and Methods

### Cell culture, reagents and animal

Human cell lines (MKN28, MKN45, MGC803, BGC823, SGC7901 and GES-1) were obtained from the Cell Biology Institute of Chinese Academy of Sciences (Shanghai, China). All cells were cultured in Dulbecco’s modified Eagle’s medium (DMEM, high glucose) (Gibco, Grand Island, NY, USA) supplemented with 10% (v/v) fetal bovine serum (FBS) (Gibco) in a humidified incubator at 37 °C with 5% CO_2_. ACh, neostigmine and AFDX-116 was obtained from Sigma (St Louis, MO, USA), AG1478, trihexyphenidyl and darifenacin were obtained from MCE (Monmouth Junction, NJ, USA). The rabbit anti-ChAT antibody was purchased from Boster Biotech (Wuhan, China), and M3R antibody was purchased from Santa Cruz (Dallas, TX, USA). Other antibodies used in this study were purchased from CellSignaling Technology (Danvers, MA, USA). BALB/c-nu mice were purchased from Da Suo laboratory animal co., LTD (Chengdu, China).

### Cell proliferation assay

To evaluate the effects of exogenous ACh on cell proliferation, both MKN45 and BGC823 cells were seeded in a 96-well plate at 1 × 10^3^ cells/well and cultured for 12 h. Then cells were treated with or without ACh in serum free medium for 3 days. To determine which muscarinic AChR subtype mediated cell growth responses, the selective antagonists for M1 AChR (trihexyphenidyl), M2/M4 AChR (AFDX-116), and M3 AChR (4-DAMP) were used. Cells were plated at 1 × 10^3^ cells/well in 96-well plates and cultured for 5 days, drugs in a range of concentrations in 100 μl were added immediately after cell plating. Every 3 days the medium plus drugs were changed. Cell viability was monitored using the Cell Counting Kit-8kit (CCK8, Dojingdo, Kumamoto, Japan) according to the manufacturer’s protocol. Briefly, 10 μl CCK8 solution was added to each well and then incubated at 37 °C for 2 h. The optical density (OD) value was read at 450 nm using a microplate reader (Bio-Rad Laboratories, CA, USA). All experiments were performed in triplicate.

### RNA interference

Sequences of small interfering RNA (siRNA) used for knockdown of M3R were designed and obtained from GenePharma (Shanghai, China). The sequences were listed as follows: M3R siRNA, 5′-CGCCUUUGUUUCCAAACAU-3′, ChAT siRNA, CCAAUCGCUGGUACGACAAGU and Control siRNA, 5′-CGTACGCGGAATACTTCGA-3′. Cells were transfected with M3R siRNA or Control siRNA using Lipofectamine 2000 (Invitrogen, Carlsbad, CA, USA), according to the manufacturer’s protocol. The knockdown efficiency was verified by western blot.

### Plate clone assay

Cells were plated at 1 × 10^4^ cells per well in 6-well plates and cultured in DMEM medium supplemented with 10% FBS. When visible colonies formed on the 48 h after plating, they were washed with phosphate-buffered saline (PBS) and fixed in 4% paraformaldehyde for 15 min, then stained with crystal violet for 15 min.

### Western blot analysis

The expression change of proteins including total and phosphorylated EGFR, EKR1/2, and AKT, and the apoptosis-related proteins in study were analyzed by western blot. Briefly, cells were harvested and lysed using RIPA lysis buffer containing protease inhibitor and phosphatase inhibitor. Then equal amounts of protein extracts were separated by 12% SDS-PAGE gel and transferred to nitrocellulose membranes using the Bio-Rad semi-dry transfer system, then the membranes were blocked in 5% non-fat dried milk for 1 h and incubated with the primary antibodies overnight at 4 °C, the membranes were incubated with secondary antibody of DyLight 800 AffiniPure goat anti-rabbit IgG or DyLight 680 AffiniPure goat anti-mouse IgG (EarthOx, CA, USA) for 2 h at room temperature. The immunoreactive bands were visualized and quantified using the ODYSSEY Infrared Imaging System (LI-ORBiosciences, Lincoln, NE, USA).

### LC-MS/MS analysis

To investigate ACh concentration released in MNK45 and BGC823 cell culture medium, 5 × 10^6^ cells in 10 ml were plated in 60 cm^2^ culture dishes. Acetylcholinesterase inhibitor neostigmine was added at a concentration of 50 μM to inhibit ACh degradation, while ChAT protein in cells were knockdown by transfected with siRNA for 48 h. After another 24 h incubation, cell suspensions were obtained and extracted by four times the volume of acetonitrile, the mixture centrifuged at 13000 rpm for 15 min at 4 °C, and 100 μl supernatants were readied to analyze. Ultra high performance LC separation was performed with an ACQUITY UPLCTM BEH C18 column (Waters Corporation, Milford, MA, USA). The mobile phases were 0.1% formic acid (A) and methanol (B), and the isocratic elution was performed with 90% A and 10% B. Waters Quattro Premier XE Mass Spectrometer coupled with ESI source was employed in the MS/MS analysis and the Masslynx V 4.1 software was used to control the UPLC-MS/MS system. The mass spectrometer was operated in the positive-ion mode with the source temperature set to 110 °C, with a cone voltage of 20 V and a capillary voltage of 2.8 kV. ACh were quantified by selective multi-reaction monitoring with a positive ionization mode.

### Immunocytochemistry of ChAT

The MKN45 and BGC823 cells were seeded on the glass slide for 24 h, and then were fixed with 4% paraformaldehyde for 15 min, following permeabilization with 1% TritonX-100 for 15 min and blocking with 1% BSA for 30 min. Then, cells were incubated with primary antibody of ChAT (1:100) overnight at 4 °C. After three times washed with PBS, the slides were incubated the biotin labeling second antibody for 20 min and stained with the chain mildew avidin peroxidase (Boster Biotech, Wuhan, China). Finally, 3,3′-diaminobenzidine (DAB) was used to color reaction and positive signals of brown granules distributed in cytoplasm were observed by optical microscope.

### *In vivo* experiments

All experiments were performed in accordance with relevant guidelines and regulations. The study was approved by the institutional animal experimental ethics committee of Sichuan University in China (Approval No. 20160017). Mice were fed in the SPF grade animal facility of West China hospital with controlled temperature and humidity. For the subcutaneously xenografted tumor models, 1 × 10^6^ MKN45 and BGC823 cells suspended in 100 μL PBS were injected s.c. into the right axillary region of 8-week-old female BALB/c nude mice. Five days after injection, 4-DAMP and darifenacin were injected i.p. at doses of 1 mg/kg/d respectively. Vehicle (PBS mixed with DMSO at 1:1) was injected in the control group. The mice were sacrificed twelve days later, the tumors were separated from the animals, tumor sizes, tumor volumes and tumor weights of each group were measured.

### Statistical analysis

Statistical analysis was performed with GraphPad Prism Software using oneway ANOVA with Tukey’s post-test and Student’s t-test. Data were expressed as the mean ± SD. *P* values < 0.05 or *P* values < 0.01 were considered statistically significant.

## Additional Information

**How to cite this article**: Yu, H. *et al*. Acetylcholine acts through M3 muscarinic receptor to activate the EGFR signaling and promotes gastric cancer cell proliferation. *Sci. Rep.* **7**, 40802; doi: 10.1038/srep40802 (2017).

**Publisher's note:** Springer Nature remains neutral with regard to jurisdictional claims in published maps and institutional affiliations.

## Supplementary Material

Supplementary Information

## References

[b1] Siegel R., Naishadham D. & Jemal A. Cancer statistics, 2013. CA Cancer J Clin 63, 11–30, doi: 10.3322/caac.21166 (2013). 23335087

[b2] Jemal A. . Global cancer statistics. CA Cancer J Clin 61, 69–90, doi: 10.3322/caac.20107 (2011). 21296855

[b3] Shiozaki H. . Evolution of gastric surgery techniques and outcomes. Chin J Cancer 35, 69, doi: 10.1186/s40880-016-0134-y (2016). 27460019 PMC4962398

[b4] van der Pijl E. M. . Characterization of neuromuscular synapse function abnormalities in multiple Duchenne muscular dystrophy mouse models. Eur J Neurosci 43, 1623–1635, doi: 10.1111/ejn.13249 (2016). 27037492

[b5] Stoiljkovic M., Kelley C., Nagy D., Leventhal L. & Hajos M. Selective activation of alpha7 nicotinic acetylcholine receptors augments hippocampal oscillations. Neuropharmacology 110, 102–108, doi: 10.1016/j.neuropharm.2016.07.010 (2016). 27422408

[b6] Peter J. . Contribution of the Cholinergic System to Verbal Memory Performance in Mild Cognitive Impairment. J Alzheimers Dis 53, 991–1001, doi: 10.3233/JAD-160273 (2016). 27340852 PMC5008225

[b7] Hall J. M. & Savage L. M. Exercise leads to the re-emergence of the cholinergic/nestin neuronal phenotype within the medial septum/diagonal band and subsequent rescue of both hippocampal ACh efflux and spatial behavior. Exp Neurol 278, 62–75, doi: 10.1016/j.expneurol.2016.01.018 (2016). 26836322 PMC4794758

[b8] Kawashima K., Fujii T., Moriwaki Y., Misawa H. & Horiguchi K. Reconciling neuronally and nonneuronally derived acetylcholine in the regulation of immune function. Annals of the New York Academy of Sciences 1261, 7–17, doi: 10.1111/j.1749-6632.2012.06516.x (2012). 22823388

[b9] Kawashima K., Fujii T., Moriwaki Y. & Misawa H. Critical roles of acetylcholine and the muscarinic and nicotinic acetylcholine receptors in the regulation of immune function. Life sciences 91, 1027–1032, doi: 10.1016/j.lfs.2012.05.006 (2012). 22659391

[b10] Winston J. H., Li Q. & Sarna S. K. Paradoxical regulation of ChAT and nNOS expression in animal models of Crohn’s colitis and ulcerative colitis. American journal of physiology. Gastrointestinal and liver physiology 305, G295–302, doi: 10.1152/ajpgi.00052.2013 (2013). 23681475 PMC3891212

[b11] Khan M. R. . M1 is a major subtype of muscarinic acetylcholine receptors on mouse colonic epithelial cells. Journal of gastroenterology 48, 885–896, doi: 10.1007/s00535-012-0718-5 (2013). 23242454

[b12] Russo P. . Cholinergic receptors as target for cancer therapy in a systems medicine perspective. Current molecular medicine 14, 1126–1138, doi: 10.2174/1566524014666141015152601 (2014). 25324001

[b13] Castillo-Gonzalez A. C. . Dysregulated cholinergic network as a novel biomarker of poor prognostic in patients with head and neck squamous cell carcinoma. BMC cancer 15, 385, doi: 10.1186/s12885-015-1402-y (2015). 25956553 PMC4435806

[b14] Spindel E. R. Cholinergic Targets in Lung Cancer. Current pharmaceutical design 22, 2152–2159, doi: 10.2174/1381612822666160127114237 (2016). 26818857 PMC4961355

[b15] Sales M. E. Muscarinic Receptors as Targets for Metronomic Therapy in Breast Cancer. Current pharmaceutical design 22, 2170–2177, doi: 10.2174/1381612822666160229115317 (2016). 26924207

[b16] Von Rosenvinge E. C. & Raufman J. P. Muscarinic receptor signaling in colon cancer. Cancers 3, 971–981, doi: 10.3390/cancers3010971 (2011). 24212649 PMC3756399

[b17] Pettersson A. . Is acetylcholine an autocrine/paracrine growth factor via the nicotinic alpha7-receptor subtype in the human colon cancer cell line HT-29? European journal of pharmacology 609, 27–33, doi: 10.1016/j.ejphar.2009.03.002 (2009). 19285065

[b18] Song P. . Acetylcholine is synthesized by and acts as an autocrine growth factor for small cell lung carcinoma. Cancer research 63, 214–221 (2003). 12517800

[b19] Wang L. . Muscarinic receptor M3 mediates cell proliferation induced by acetylcholine and contributes to apoptosis in gastric cancer. Tumour biology: the journal of the International Society for Oncodevelopmental Biology and Medicine 37, 2105–2117, doi: 10.1007/s13277-015-4011-0 (2016). 26346168

[b20] Shah N., Khurana S., Cheng K. & Raufman J. P. Muscarinic receptors and ligands in cancer. American journal of physiology. Cell physiology 296, C221–232, doi: 10.1152/ajpcell.00514.2008 (2009). 19036940 PMC2643856

[b21] Williams C. L. Muscarinic signaling in carcinoma cells. Life sciences 72, 2173–2182, doi: 10.1016/S0024-3205(03)00080-8 (2003). 12628476

[b22] Yin J. & Yu F. S. ERK1/2 mediate wounding- and G-protein-coupled receptor ligands-induced EGFR activation via regulating ADAM17 and HB-EGF shedding. Investigative ophthalmology & visual science 50, 132–139, doi: 10.1167/iovs.08-2246 (2009). 18658095 PMC3656386

[b23] Bapat A. A., Hostetter G., Von Hoff D. D. & Han H. Perineural invasion and associated pain in pancreatic cancer. Nature reviews. Cancer 11, 695–707, doi: 10.1038/nrc3131 (2011). 21941281

[b24] Marchesi F., Piemonti L., Mantovani A. & Allavena P. Molecular mechanisms of perineural invasion, a forgotten pathway of dissemination and metastasis. Cytokine Growth Factor Rev 21, 77–82, doi: 10.1016/j.cytogfr.2009.11.001 (2010). 20060768

[b25] Jobling P. . Nerve-Cancer Cell Cross-talk: A Novel Promoter of Tumor Progression. Cancer research, doi: 10.1158/0008-5472.CAN-14-3180 (2015). 25795709

[b26] Magnon C. . Autonomic nerve development contributes to prostate cancer progression. Science 341, 1236361, doi: 10.1126/science.1236361 (2013). 23846904

[b27] Zhao C. M. . Denervation suppresses gastric tumorigenesis. Science translational medicine 6, 250ra115, doi: 10.1126/scitranslmed.3009569 (2014). PMC4374618 25143365

[b28] Paleari L., Grozio A., Cesario A. & Russo P. The cholinergic system and cancer. Seminars in cancer biology 18, 211–217, doi: 10.1016/j.semcancer.2007.12.009 (2008). 18262434

[b29] Spindel E. R. Muscarinic receptor agonists and antagonists: effects on cancer. Handbook of experimental pharmacology, 451–468, doi: 10.1007/978-3-642-23274-9_19 (2012). 22222710 PMC3604886

[b30] Peretto I., Petrillo P. & Imbimbo B. P. Medicinal chemistry and therapeutic potential of muscarinic M3 antagonists. Medicinal research reviews 29, 867–902, doi: 10.1002/med.20158 (2009). 19399831

[b31] Jiang Y., Li Y. R., Tian H., Ma M. & Matsunami H. Muscarinic acetylcholine receptor M3 modulates odorant receptor activity via inhibition of beta-arrestin-2 recruitment. Nature communications 6, 6448, doi: 10.1038/ncomms7448 (2015). PMC4372811 25800153

[b32] Raufman J. P. . Muscarinic receptor subtype-3 gene ablation and scopolamine butylbromide treatment attenuate small intestinal neoplasia in Apcmin/+mice. Carcinogenesis 32, 1396–1402, doi: 10.1093/carcin/bgr118 (2011). 21705482 PMC3165126

[b33] Wu J. . High expression of M3 muscarinic acetylcholine receptor is a novel biomarker of poor prognostic in patients with non-small cell lung cancer. Tumour biology: the journal of the International Society for Oncodevelopmental Biology and Medicine 34, 3939–3944, doi: 10.1007/s13277-013-0982-x (2013). 23838802

[b34] Feng Y. J., Zhang B. Y., Yao R. Y. & Lu Y. Muscarinic acetylcholine receptor M3 in proliferation and perineural invasion of cholangiocarcinoma cells. Hepatobiliary & pancreatic diseases international: HBPD INT 11, 418–423, doi: 10.1016/S1499-3872(12)60201-X (2012). 22893470

[b35] Xie G., Cheng K., Shant J. & Raufman J. P. Acetylcholine-induced activation of M3 muscarinic receptors stimulates robust matrix metalloproteinase gene expression in human colon cancer cells. American journal of physiology. Gastrointestinal and liver physiology 296, G755–763, doi: 10.1152/ajpgi.90519.2008 (2009). 19221016 PMC2670666

[b36] Yang T. . MACC1 mediates acetylcholine-induced invasion and migration by human gastric cancer cells. Oncotarget 7, 18085–18094, doi: 10.18632/oncotarget.7634 (2016). 26919111 PMC4951273

[b37] Song P. . M3 muscarinic receptor antagonists inhibit small cell lung carcinoma growth and mitogen-activated protein kinase phosphorylation induced by acetylcholine secretion. Cancer research 67, 3936–3944, doi: 10.1158/0008-5472.can-06-2484 (2007). 17440109

