# Supplementary material for: Acetylcholine acts through M3 muscarinic receptor to activate the EGFR signaling and promotes gastric cancer cell proliferation
Source: Sci Rep. 2017 Jan 19;7:40802. doi: 10.1038/srep40802 (PMC5244394; doi:10.1038/srep40802)
Supplement: Supplementary Information [file srep40802-s1.pdf]

Acetylcholine acts through M3 muscarinic receptor to activate the EGFR signaling and promotes gastric cancer cell proliferation

Huangfei Yu[1,*], Hongwei Xia[1,*], Qiulin Tang[1], Huanji Xu[1], Guoqing Wei[1], Ying Chen[1], Xinyu Dai[1], Qiyong Gong[2]& Feng Bi[1]

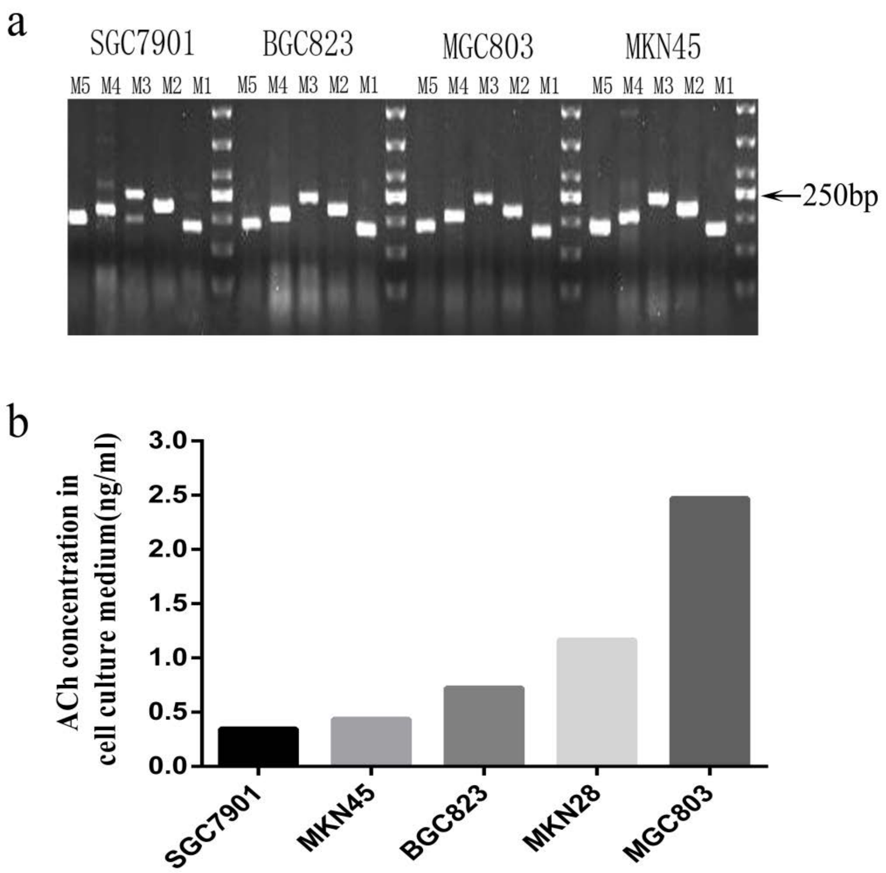

Supplemental Fig.1. The expression of muscarinic receptor subtypes and the basic ACh content of culture medium in gastric cancer cells . **a** M1~M5 receptor's mRNA in indicated cells were detected by polymerase chain reaction and semi-quantity analyzed. **b** The basic contents of ACh in serum free medium of MKN28, MKN45, MGC803, BGC823 and SGC7901 cells were measured by LC-MS/MS.

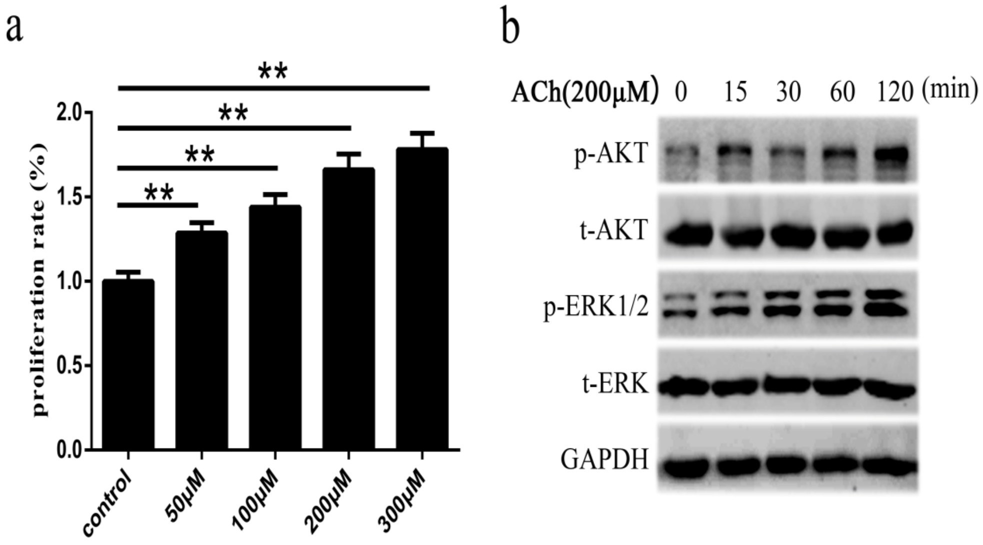

Supplemental Fig.2. ACh promotes cell proliferation and stimulates phosphorylation of ERK1/2 and AKT in SGC7901 cells. **a** SGC7901 cells were incubated with ACh for 72 h at indicated concentrations. CCK-8 assay was used to determine the proliferation of cancer cells. **b** Western blot was performed to show expression changes of the indicated proteins after treating cells with 200 μM ACh for 15 min to 120 min.(** $P < 0.01$).

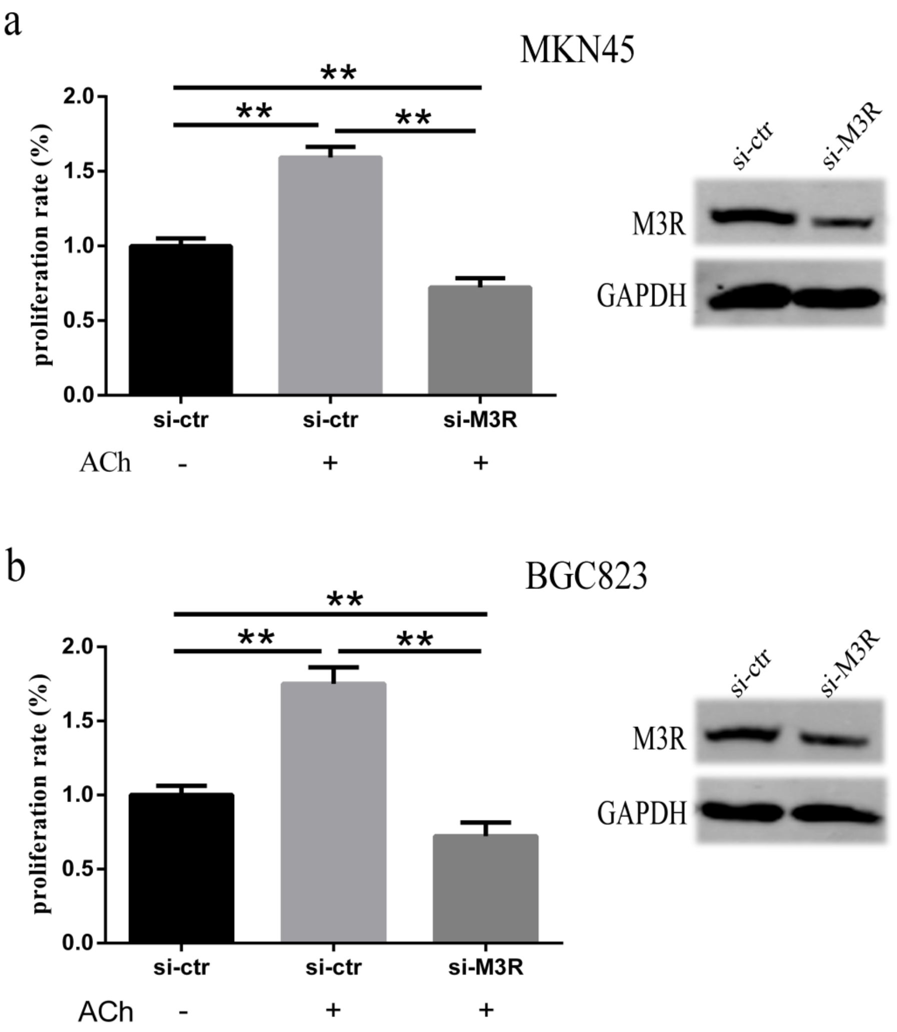

Supplemental Fig.3. Knockdown M3R expression reversed ACh-induced cell proliferation in MKN45 and BGC823 cells. 200μM ACh was used to stimulate MKN45(**a**)and BGC823(**b**) cells after cells were transfected with M3R siRNA and control siRNA for 2 days, cell proliferation of each group was determined by using CCK-8 assay, and the knockdown efficiency was verified by western blot.(** $P < 0.01$).

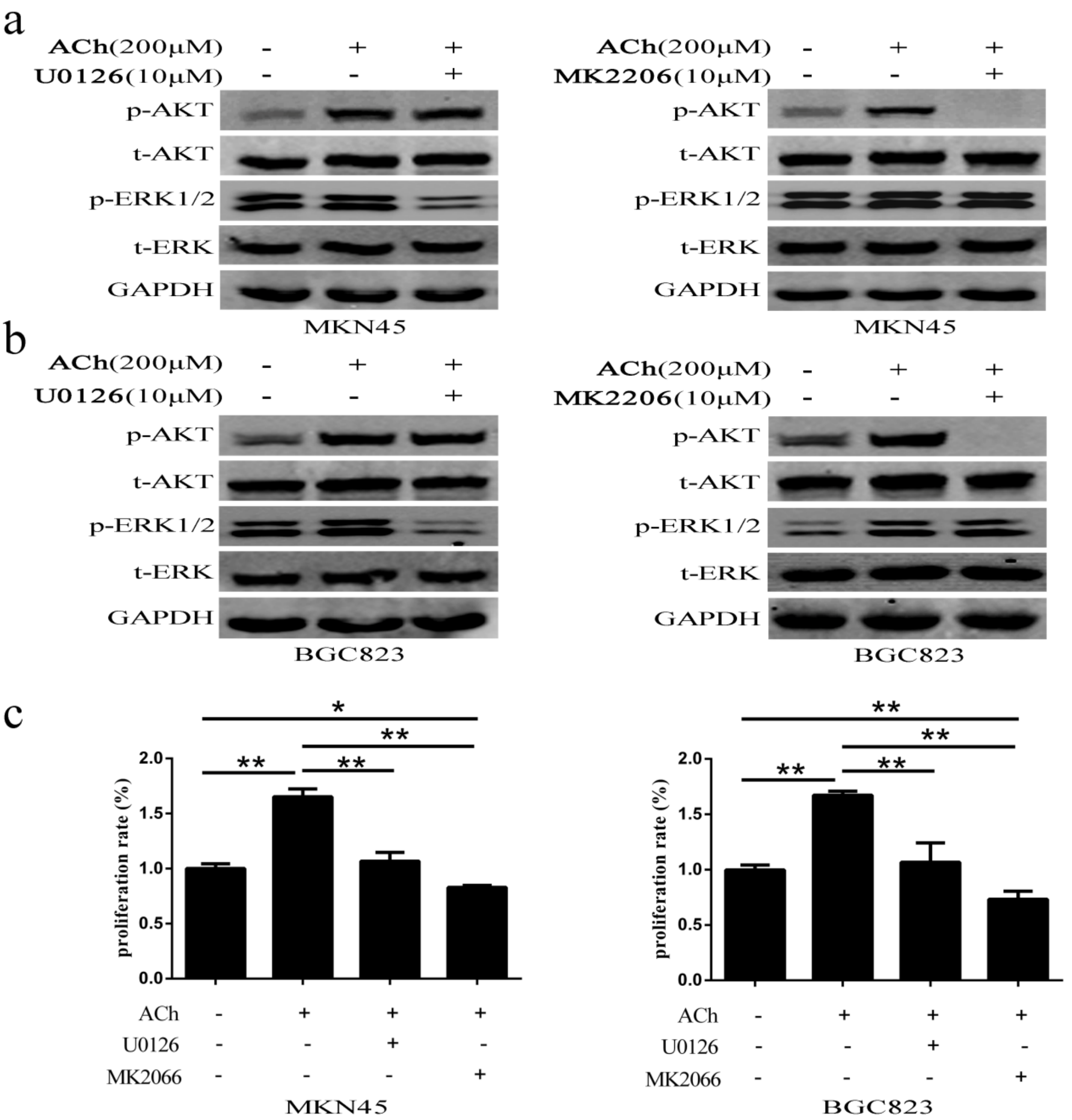

Supplemental Fig.4. ERK inhibitor U0126 and AKT inhibitor MK2206 block the ACh-induced effects in MKN45 and BGC823 cells. **a** After adding 10μM ERK specific inhibitor U0126 or AKT specific inhibitor MK2206 2 h prior to ACh addition, protein changes in MKN45 and BGC823(**b**) cells were analyzed by western blot. **c** U0126 or MK2206 was added 2 h before ACh addition, CCK-8 assay was used to study the role of ERK and AKT in ACh induced cell proliferation. (* $P < 0.05$; ** $P < 0.01$).

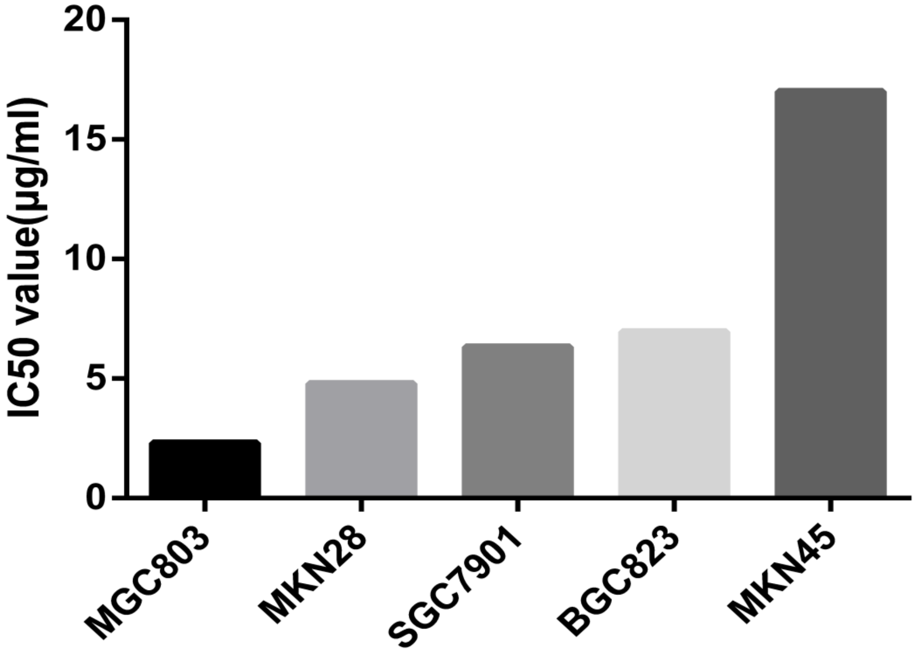

Supplemental Fig.5. IC50 value of 5-Fu in different gastric cancer cell lines. Indicated gastric cancer cells were plated at 5000 cells per well in 96 well plate and cultured for 24 h, 5-Fu was added at the concentration of 0.3125μg/ml to 40μg/ml in geometric dilution method, CCK-8 assay was used to determine the cell viability after 24 h incubation and IC50 value of each groups were calculated.
